# Pharmacological inhibition of GSK-3 in a guinea pig model of LPS-induced pulmonary inflammation: I. Effects on lung remodeling and pathology

**DOI:** 10.1186/1465-9921-14-113

**Published:** 2013-10-23

**Authors:** Hoeke A Baarsma, Sophie Bos, Herman Meurs, Kim H Visser, Marieke Smit, Annemie MWJ Schols, Ramon C Langen, Huib AM Kerstjens, Reinoud Gosens

**Affiliations:** 1 Department of Molecular Pharmacology, University of Groningen, Antonius Deusinglaan 1, 9713 AV Groningen, The Netherlands; 2 Department of Pulmonology, University Medical Center Groningen, University of Groningen, Groningen, The Netherlands; 3 Groningen Research Institute for Asthma and COPD, University of Groningen, Groningen, The Netherlands; 4 Respiratory Medicine, Maastricht University, Groningen, The Netherlands

## Abstract

**Background:**

Glycogen synthase kinase-3 (GSK-3) is a constitutively active kinase that regulates multiple signalling proteins and transcription factors involved in a myriad of cellular processes. The kinase acts as a negative regulator in β-catenin signalling and is critically involved in the smad pathway. Activation of both pathways may contribute to pulmonary features of chronic obstructive pulmonary disease (COPD).

**Methods:**

In the present study, we investigated the effect of the selective GSK-3 inhibitor SB216763 on pulmonary pathology in a guinea pig model of lipopolysaccharide (LPS)-induced COPD. Guinea pigs were instilled intranasally with LPS or saline twice weekly for 12 weeks and pre-treated with either intranasally instilled SB216763 or corresponding vehicle 30 min prior to each LPS/saline challenge.

**Results:**

Repeated LPS exposures activated β-catenin signalling, primarily in the airway epithelium and submucosa. LPS also induced pulmonary inflammation and tissue remodelling as indicated by inflammatory cell influx, increased pulmonary fibronectin expression and enhanced small airway collagen content. Inhibition of GSK-3 by SB216763 did not affect LPS-induced inflammatory cell influx, but prevented the small airway remodelling and, unexpectedly, inhibited the activation of β-catenin *in vivo*. LPS or SB216763 treatment had no effect on the airway smooth muscle content and alveolar airspace size. However, GSK-3 inhibition prevented LPS-induced right ventricle hypertrophy.

**Conclusions:**

Our findings indicate that GSK-3 inhibition prevents LPS-induced pulmonary pathology in guinea pigs, and that locally reduced LPS-induced β-catenin activation appears in part to underlie this effect.

## Introduction

Glycogen synthase kinase-3 (GSK-3) is a ubiquitously expressed serine/threonine kinase, occurring in the two closely related isoforms GSK-3α and GSK-3β which share high homology in their kinase domains [1-3]. Originally, GSK-3 was discovered for its role in glucose metabolism by regulating glycogen synthase activity [3,4]. Over the years, interest in GSK-3 signalling has increased as it became apparent that this kinase regulates various physiological pathways involved a wide array of processes, including protein synthesis, cell differentiation, apoptosis and cell survival [5,6]. Currently, over fifty putative substrates have been identified including structural proteins, various intracellular signalling intermediates and transcription factors [6]. For instance, GSK-3 is critically involved as a negative regulator in β-catenin signalling and in the regulation of smad-dependent signalling [5,6]. Both these pathways are important in developmental processes and may be activated during pathological conditions in the lungs [7-10].

In the β-catenin signalling pathway, GSK-3 is the primary kinase that regulates cellular expression of the transcriptional co-activator β-catenin by phosphorylation, thereby targeting it for proteasomal degradation [11]. In pulmonary fibroblasts, we recently demonstrated that the pro-fibrotic mediator transforming growth factor-β (TGF-β) induces an inhibitory phosphorylation of GSK-3 and activates β-catenin signalling, which in turn contributed to myofibroblast differentiation and extracellular matrix deposition by these cells [12]. Interestingly, β-catenin activation and extracellular matrix deposition were enhanced in fibroblasts of individuals with chronic obstructive pulmonary disease (COPD) [12].

Despite its inhibitory role in β-catenin signalling, GSK-3 is required for fibrosis in mice [13]. In line with this, we have shown in human pulmonary fibroblasts that GSK-3 is required for myofibroblast differentiation and matrix protein expression [14]. Mechanistically, this is explained by activation of cyclic AMP response element binding protein (CREB) signalling in response to GSK-3 inhibition, which can attenuate smad-dependent transcriptional responses. It appears therefore that GSK-3 inhibition plays a dual role in pathological tissue remodelling. On one hand, GSK-3 is the main negative regulator of β-catenin of which increased activation is associated with fibroproliferative diseases, whereas on the other hand GSK-3 inhibition may attenuate smad-dependent gene transcription and fibrotic responses. This dual role may be tightly controlled by the subcellular localization of GSK-3, as only the GSK-3 pool that is associated with the multi-protein destruction complex consisting of axin, casein kinase I and APC is involved in β-catenin signalling [15,16].

In the present study, we investigated the effect of GSK-3 inhibition on β-catenin activation, inflammation and matrix protein expression in response to lipopolysaccharide (LPS)-, using the selective inhibitor 3-(2,4-dichlorophenyl)-4-(1-methyl-1H-indol-3-yl)-1H-pyrrole-2,5-dione (SB216763). LPS is an endotoxin in the outer membrane of gram negative bacteria that is present as a contaminant in environmental pollution, organic dusts and cigarette smoke, which are all factors that have been associated with COPD development [17,18]. Furthermore, bacterial endotoxins may contribute to COPD exacerbations [19]. Accordingly, we and others have previously demonstrated that LPS can induce pulmonary and extrapulmonary pathological features resembling COPD pathophysiology in various animal models [20,21].

## Materials and methods

### Animals

Outbred, male, specified pathogen-free Dunkin Hartley guinea pigs (Harlan, Heathfield, United Kingdom) were used. All protocols describes in this study were approved by the University of Groningen Committee for Animal Experimentation.

### Experimental protocol

Thirty-six guinea pigs were randomly assigned to four experimental groups, composed of vehicle treated, saline challenged (n = 9); vehicle treated; LPS-challenged (n = 9), SB216763 treated, saline challenged (n = 9) and SB216763 treated; LPS-challenged (n = 9). Guinea pigs were treated twice weekly for 12 consecutive weeks by intranasal instillation of 100 μL SB216763 (2 mM in 10% v/v DMSO in sterile saline) or vehicle (10% DMSO v/v in sterile saline). After the intranasally instilled solution was aspirated, the animals were kept in an upright position for an additional 2 minutes, to allow sufficient spreading of the fluid throughout the lungs. Thirty minutes after the instillations of SB216763 or vehicle, the animals were intranasally instilled with 100 μL LPS (10 mg/ml in sterile saline) or sterile saline. We have previously confirmed the suitability of these protocols for efficient pulmonary delivery of intranasally instilled solutions using, among others, Evan’s Blue dye as a control [20]. Twenty four hours after the last instillation, the guinea pigs were killed by experimental concussion, followed by rapid exsanguination. The heart and lungs of each animal were resected and kept on ice for immediate further processing.

### Tissue processing and histological analyses

The left lung lobe was inflated and fixed with formalin at a constant pressure of 25 cm H_2_O for 24 hours. The formalin fixed lungs were embedded in paraffin and subsequently cut in tissue-sections of 4 μm. The mean linear intercept (LMI), a measure for alveolar airspace size, was determined in tissue-sections stained with haematoxylin and eosin. The LMI was determined as described previously [20], by using 20-25 photo-microscopic images (magnification 200×) per animal. The LMI analysis was performed twice by two individuals in a blinded manner. For evaluation of airway collagen, the tissue-sections (4 μm) were stained with Sirius Red and counterstained with haematoxylin. Airways were digitally photographed (100-200 × magnification) and using ImageJ software, each image was split into RGB channels. The green channel images were used for further analysis and converted to binary images using the threshold function setting the threshold value identical for all images. The positively stained area in the airway wall, from adventitial border to the basement membrane, was digitally quantified in at least 2 airways per animal. The airway collagen area was then normalized to the squared basement membrane length. The analysis was done in a blinded manner.

The upper right lung lobe was immediately frozen in liquid nitrogen after resection. Transverse frozen sections (4 μM) of the right lung lobe were used for immunohistochemical analysis. The smooth muscle area was identified using immunohistochemical staining for smooth-muscle-specific myosin heavy chain (sm-MHC; dilution 1:100, Neomarkers, Fremont, CA, USA). To identify granulocytes, sections were stained with diaminobenzidine (0.3 mg/ml). The specific primary antibodies were visualized by using horseradish peroxidase (HRP)-linked secondary antibodies, followed by a diaminobenzidine staining (0.1 mg/ml for sm-MHC). Sections were counter stained with haematoxylin. The airways within sections were digitally photographed (200 × magnification) and classified as cartilaginous or non-cartilaginous. All immunohistochemical analyses were performed using ImageJ software. Per animal, at least 2 lung sections were analysed per staining, each section containing 2-5 airways. The sm-MHC positively stained area was digitally quantified and normalized to the squared basement membrane length. The number of inflammatory cells within 50 μm distance from the airway lumen was quantified and expressed relative to basement membrane length. All analyses were done in a blinded manner.

For identification of activated β-catenin, sections were stained for non-phosphorylated β-catenin (dilution 1:50; Active β-catenin; ABC clone 8E7, Millipore, Amsterdam, The Netherlands). The specific primary antibodies were visualized by using Cy3-conjugated secondary antibodies and analysed using an Olympus AX70 microscope equipped with digital image capture system (ColorView Soft System with Olympus U CMAD2 lens). Sections were counter stained with Hoechst 3342.

### Immunoblotting

The lower right lung lobe was used for protein analysis by immunoblotting. Lung homogenates were prepared by pulverizing the frozen tissue under liquid nitrogen, after which 300 mg tissue was sonicated in 1 ml of ice-cold radio-immunoprecipation (RIPA) buffer supplemented with protease and phosphatase inhibitors (composition: Tris–HCl 50.0 mM, NaCl 150.0 mM, EDTA 1.0 mM, Na3VO_4_ 1.0 mM, NaF 1.0 mM, Na-deoxycholate 0.25% and 1% Igepal (NP-40), supplemented with 5 mM β-glycerophosphate, 10 μg/ml leupeptin, 10 μg/ml aprotinin and 10 μg/ml pepstatin; at pH 7.4).

Equal amounts of protein (50 μg/lane) were subjected to electrophoresis on polyacrylamide gels, transferred to nitrocellulose membranes and analysed for the proteins of interest using specific primary and HRP-conjugated secondary antibodies. By using enhanced chemiluminescence reagents, bands were recorded in the G:BOX iChemi gel documentation system equipped with GeneSnap image acquisition software (Syngene; Cambridge; UK). Band intensities were quantified by densitometry using GeneTools analysis software (Syngene; Cambridge; UK).

### Antibodies and reagents

The mouse anti-smooth-muscle-specific myosin heavy chain (sm-MHC) antibody was from Neomarkers (Fremont, CA, USA). Horseradish peroxidase (HRP)-conjugated goat anti-mouse antibody, HRP-conjugated goat anti-rabbit antibody, HRP-conjugated rabbit anti-goat antibody and lipopolysaccharides (LPS) from *Escherichia coli* (055:B5) were purchased from Sigma (St. Louis, MO, USA). Cy3 conjugated secondary antibodies were obtained from Jackson ImmunoResearch (West Grove PA, USA). Mouse anti-GSK-3 antibody, goat anti-fibronectin (C20) antibody and mouse anti-glyceraldehyde-3-phosphate dehydrogenase (GAPDH) antibody were obtained from Santa Cruz Biotechnology (Santa Cruz, CA, USA). Rabbit anti-phospho-Ser9/21-GSK-3 antibody was from Cell Signaling Technology (Beverly, MA, USA). Mouse anti-total β-catenin antibody was from BD Biosciences (San Jose, CA, USA). Mouse anti-non-Ser37/Thr41-phosphorylated-β-catenin antibody (clone 8E7) was from Millipore (Amsterdam, the Netherlands). The selective GSK-3 inhibitor 3-(2,4-Dichlorophenyl)-4-(1-methyl-1*H*-indol-3-yl)-1*H*-pyrrole-2,5-dione (SB216763) was obtained from Tocris Cookson (Bristol, UK). Recombinant human TGF-β_1_ was from R&D systems (Abingdon, UK). All other chemicals were of analytical grade.

### Statistical analysis

Data represent means ± S.E.M, from *n* separate experiments. Statistical significance of differences was evaluated by one-way or two-way ANOVA, where appropriate, followed by a Newman-Keuls multiple comparison test. Differences were considered to be statistically significant when p < 0.05.

## Results

### Effect of repeated LPS instillation and GSK-3 inhibition on extracellular matrix turnover

**Figure 1:**
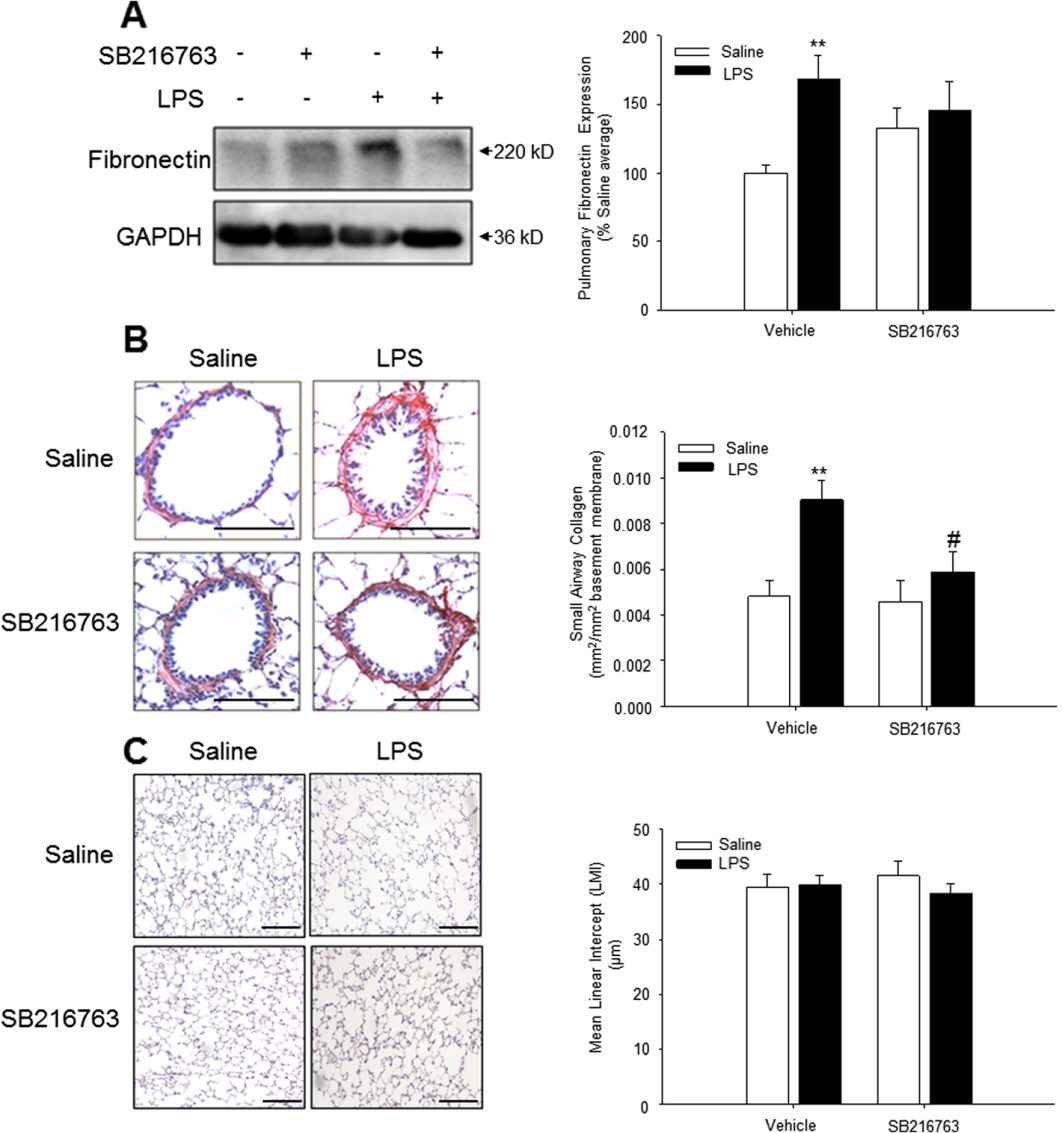
**Effect of repeated intranasal LPS challenge and treatment with the selective GSK-3 inhibitor SB216763 on extracellular matrix deposition in the lung. (A)** Expression of fibronectin was evaluated in whole lung homogenates 24 hours after the last challenge by immunoblotting using specific antibodies. Equal protein loading was verified by the analysis of GAPDH. Effects of repeated LPS challenge and SB216763 treatment on fibronectin expression were quantified by densitometry, representing mean ± s.e.m. of 9 animals per group. **(B)** Histological staining of the extracellular matrix protein collagen using Sirius Red. The non-cartilaginous airways were digitally photographed (100-200 × magnification) and analysed by using ImageJ software. Effects of repeated LPS challenge and SB216763 treatment on airway collagen expression were quantified, representing mean ± s.e.m. of 9 animals per group. **(C)** The mean linear intercept (LMI), a measure for alveolar airspace size, was determined by staining the tissue-sections with haematoxylin and eosin. Data represent means ± s.e.m. of 9 animals per group. **p < 0.01 compared to control group and ^#^p < 0.05 compared to LPS treated animals. Scale bar = 200 μm.

First, we evaluated the effects of repeated LPS instillation and SB216763 treatment on airway fibrosis. To this aim, the lungs of the guinea pigs were analysed for the expression of the extracellular matrix proteins fibronectin and collagen. Repeated LPS instillation caused a significant up regulation of fibronectin expression in whole lung homogenates (Figure 1A). Pulmonary fibronectin expression appeared to be up regulated by GSK-3 inhibition; however, this was not statistically significant. Interestingly, fibronectin expression after repeated LPS instillation and treatment with SB216763 was similar to the effect of treatment with just SB216763. Statistical analysis revealed a trend towards a negative interaction between the effect of SB216763 and of LPS (p = 0.052, determined by two-way ANOVA).

Next, we determined the expression of collagen in non-cartilaginous airways by quantitative analysis of Sirius Red staining in these airways. Similar to the increase in pulmonary fibronectin expression, repeated LPS instillation increased small airway collagen content by 1.88 ± 0.18 fold compared to the average of the saline treated animals (Figure 1B). Topical treatment of the airways with intranasally instilled SB216763 fully inhibited the LPS-induced increase in collagen deposition in the walls of the small airways, whereas the selective GSK-3 inhibitor did not affect the collagen content in saline treated animals (Figure 1B).

Emphysema, a pathological feature defined by the loss of the alveolar structure and increased parenchymal airspaces may be caused by tissue destruction in combination with an impaired repair process within the parenchyma [17]. To evaluate the effect of GSK-3 inhibition on the size of the alveolar airspaces, LMI was determined in paraffin-embedded lung sections. Repeated LPS instillation for 12 weeks did not significantly affect the LMI and, more importantly, inhibition of GSK-3 by SB216763 did not affect the size of the alveolar airspaces in either saline- or LPS-instilled animals (Figure 1C). Collectively, this indicates that repeated instillation of LPS induces alterations in pulmonary extracellular matrix expression and that inhibition of GSK-3 is beneficial in attenuating small airway fibrosis without affecting alveolar airspace size.

### Effects of repeated LPS instillation and GSK-3 inhibition on airway smooth muscle content

**Figure 2:**
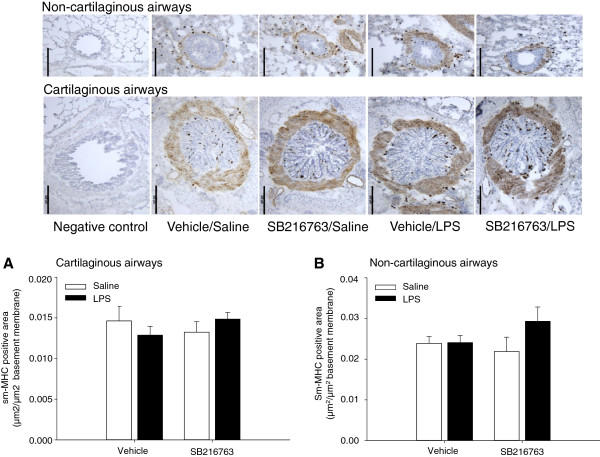
**Repeated LPS instillation and pharmacological inhibition of GSK-3 by SB216763 do not affect airway smooth muscle content.** Immunohistological analysis of sm-MHC positive area in **(A)** large (cartilaginous) and **(B)** small (non-cartilaginous) airways. Effects of repeated LPS challenge and SB216763 treatment on airway smooth muscle sm-MHC expression were quantified. Data represent means ± s.e.m. of 9 animals per group. Scale bar = 200 μm.

Published findings indicate that growth factor induced inhibition of GSK-3 promotes airway smooth muscle cell proliferation and hypertrophy [22,23]. Therefore, the airway smooth muscle content in cartilaginous and non-cartilaginous airways was determined by staining transverse frozen lung sections for the specific marker smooth-muscle myosin heavy chain (sm-MHC). Representative photomicrographs of serial lung sections containing the larger (cartilaginous) and small (non-cartilaginous) airways are shown in Figure 2. Morphometric analysis revealed that neither repeated LPS instillation nor SB216763 treatment affected the smooth muscle content in either the cartilaginous or the non-cartilaginous airways (Figure 2).

### Effects of repeated LPS instillation and GSK-3 inhibition on right ventricle hypertrophy

**Figure 3:**
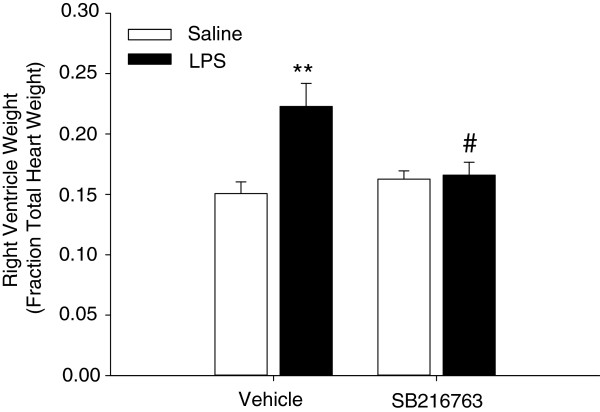
**Right ventricle hypertrophy after repeated intranasal LPS instillation is attenuated by GSK-3 inhibition.** Effect of repeated LPS instillation and GSK-3 inhibition by SB216763 on right ventricle hypertrophy. Effects of repeated LPS challenge and SB216763 treatment on size of right ventricle were quantified as right ventricle weight over total heart weight, representing mean ± s.e.m. of 9 animals per group. **p < 0.01 compared to control group and ^#^p < 0.05 compared to LPS treated animals.

A well-known co-morbidity in COPD is the occurrence of pulmonary hypertension resulting in alterations in structure and function of the right ventricle of the heart [24]. Repeated LPS challenge induced right ventricle hypertrophy in the guinea pigs as indicated by a significant 1.48 ± 0.13-fold increase in the ratio of right ventricle weight over total heart weight compared to saline treated animals (Figure 3). SB216763 fully prevented the LPS-induced right ventricle hypertrophy; whereas the selective GSK-3 inhibitor did not have an effect in saline treated animals (Figure 3).

### GSK-3 inhibition does not affect LPS-induced airway inflammation

**Figure 4:**
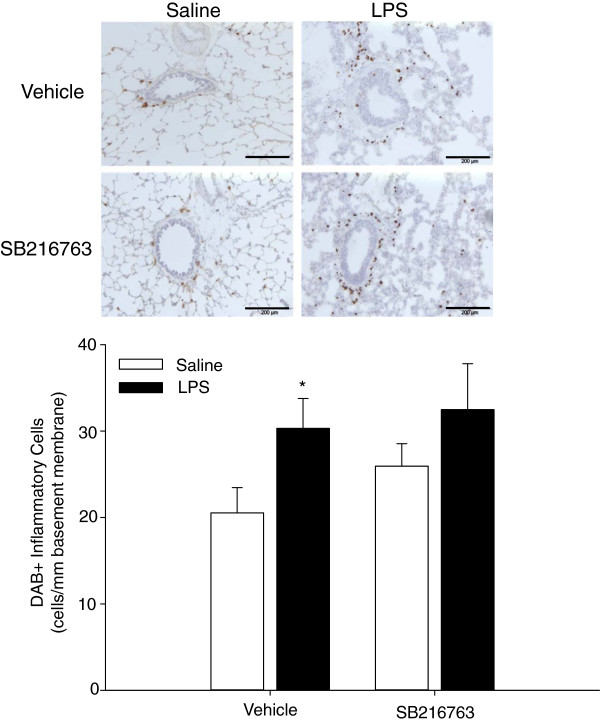
**GSK-3 inhibition does not inhibit LPS-induced pulmonary inflammation.** Effect of repeated LPS instillation and GSK-3 inhibition by SB216763 on inflammatory cell infiltration in the airways. Cells within 50 μm of the basement membrane were quantified and expressed relative to basement membrane length, representing mean ± s.e.m. of 9 animals per group. *p < 0.05 compared to control group. Scale bar = 200 μm.

We previously reported that repeated LPS exposures induce an increase in infiltrated inflammatory cells in the airways, consisting mostly of neutrophils [20]. Also, GSK-3 inhibition was previously reported to have anti-inflammatory effects *in vitro* and *in vivo*. Indeed, in the current study, repeated LPS challenges induced an increase in peribronchial inflammation, which was most profound in the non-cartilaginous airways. Surprisingly however, treatment with SB216763 did not affect the presence of inflammatory cells in and surrounding the airway wall in saline challenged animals and had no effect on the peribronchial inflammation seen after LPS challenge (Figure 4). This suggests that the effects of GSK-3 inhibition on airway fibrosis were not via anti-inflammatory effects in this model. Further evaluation of the inflammatory response was not possible unfortunately due to the lack of species cross-reactivity of commercially available tools.

### Effects of repeated LPS instillation and GSK-3 inhibition on β-catenin activation

**Figure 5:**
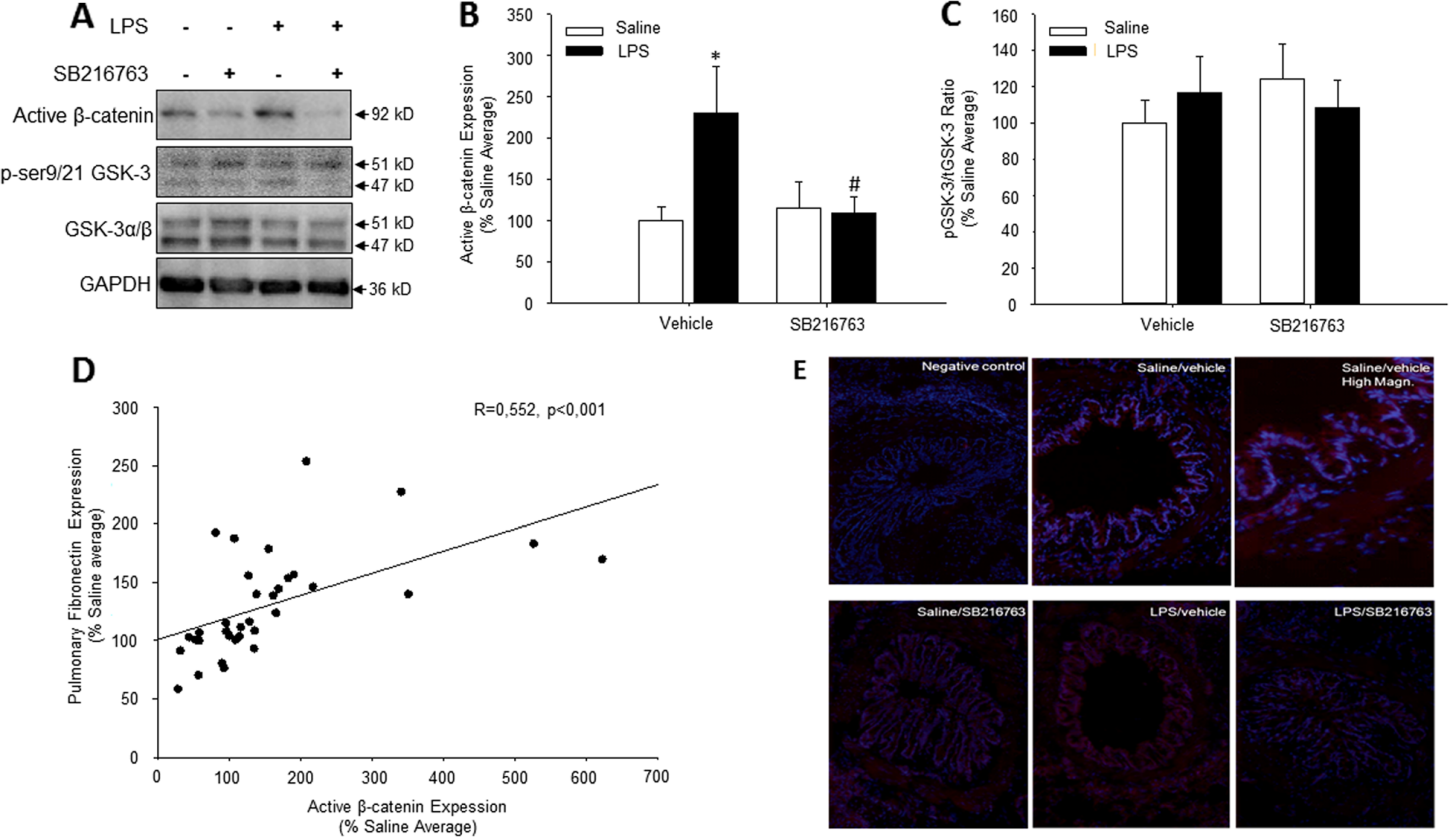
**Activation of β-catenin in response to repeated intranasal LPS challenge is prevented by treatment with the selective GSK-3 inhibitor SB216763. (A)** Expression of active β-catenin, phosphorylated GSK-3 (ser9/21 GSK-3) and total GSK-3 was evaluated in whole lung homogenates 24 hours after the last challenge by immunoblotting using specific antibodies. Equal protein loading was verified by the analysis of GAPDH. **(B,C)** Responses of repeated LPS challenge and SB216763 treatment on active β-catenin expression **(B)** and GSK-3 phosphorylation **(C)** were quantified by densitometry, representing mean ± s.e.m. of 9 animals per group. **(D)** Correlation between pulmonary expression of fibronectin (data from Figure 1) and active β-catenin in all guinea pigs. R = 0.552; p < 0.001. **(E)** Immunofluorescence analysis of active β-catenin (red) in large airways counterstained with Hoechst 3342 to stain nuclei (blue). *p < 0.05 compared to control group and ^#^p < 0.05 compared to LPS treated animals.

We aimed to gain more mechanistic insight into the reduced airway fibrosis we observed after GSK-3 inhibition. We investigated the activation of β-catenin signalling in whole lung homogenates in response to repeated intranasal instillation of LPS. The endotoxin LPS clearly induced the expression of active, non-Ser37/Thr41-phosphorylated β-catenin in whole lung homogenates compared to the saline treated animals (Figure 5A and B). Fibrotic changes in the lungs may be due to activation of β-catenin signalling [8,12,25]. Therefore, we analysed the correlation between active β-catenin expression and the amount of fibronectin in whole lung homogenate (Figure 5D). A significant linear correlation (R = 0.552; p < 0.001) exists between the presence of active β-catenin and pulmonary fibronectin expression (for both parameters the average of vehicle/saline treated animals was set to 100%). Immunofluorescence analysis revealed that activated β-catenin was expressed primarily in the airway epithelium and the airway smooth muscle (Figure 5E).

The selective GSK-3 inhibitor SB216763 did not significantly affect the expression of the active form of β-catenin compared to that in saline treated animals (Figure 5A and B). Unexpectedly however, selective inhibition of GSK-3 attenuated the LPS-induced expression of β-catenin to levels comparable to those in saline treated animals (Figure 5A, B and E). GSK-3 is considered a constitutively active kinase, which is inhibited upon serine phosphorylation (i.e. ser9 on GSK-3β and ser21 on GSK-3α). The phospho-serines act as a pseudo-substrate for the kinase itself, thereby competitively preventing the accessibility of other substrates to the active site of the kinase [6]. LPS did not induce the inhibitory serine phosphorylation of GSK-3 in whole lung homogenates. Treatment with SB216763 had no effect on GSK-3 phosphorylation either in saline or LPS-exposed animals (Figure 5A and C).

## Discussion

In this study, we demonstrate that glycogen synthase kinase-3 (GSK-3) signalling significantly contributes to the development of pathological features in response to repeated LPS exposures in guinea pigs. Repeated intranasal LPS instillation induced the activation of β-catenin signalling and remodelling with an increase in pulmonary fibronectin expression and enhanced collagen content in the smaller, non-cartilaginous airways. Unexpectedly, pharmacological inhibition of GSK-3 by topical administration of the small molecule inhibitor SB216763 prevented the LPS-induced activation of β-catenin signalling. Further, *in vivo* treatment with SB216763 prevented the small airway remodelling, and right ventricle hypertrophy, and had no detrimental effect on alveolar airspace size or airway smooth muscle content. Collectively, these data indicate that GSK-3 plays a paradoxical dual role in β-catenin signalling and may be a beneficial therapeutic target.

Airway fibrosis is a characteristic feature of COPD, which contributes to airway wall thickening and airflow limitation [26]. We demonstrate that repeated LPS instillation resulted in increased expression of the extracellular matrix proteins fibronectin and collagen. The pulmonary expression of fibronectin significantly correlated to the protein level of activated β-catenin, which was predominantly present in the epithelial cells lining the airways and the submucosa. Further analysis revealed that LPS also induces small airway fibrosis as determined by collagen content in the non-cartilaginous airways. We and others have previously shown that pulmonary fibronectin expression is regulated by canonical WNT/β-catenin signalling [25,27,28]. Activation of β-catenin is important in normal wound healing, however aberrant activation of this transcriptional co-activator has been associated with various fibroproliferative diseases, including chronic lung diseases [7,8,29]. β-Catenin may directly be responsible for the transcription of *fibronectin*, via its interaction with T-cell-factor/lymphoid enhancer factor (TCF/LEF) transcription factors [27]. Furthermore, β-catenin may also increase fibronectin expression in an indirect manner by up regulating TGF-β expression and subsequent activation of smad signalling [30]. Thus, β-catenin appears to play an important role in airway fibrosis, including that seen in our animal model.

Paradoxically, pharmacological inhibition of GSK-3 by topical administration of SB216763 prevented the LPS-induced collagen and fibronectin expression but had no effect on the inflammatory response suggesting that this is a direct effect on matrix protein expression. These findings are paradoxical as GSK-3 is a negative regulator of β-catenin expression in fibroblasts [12]. Furthermore, GSK-3 is a well-known suppressor of epithelial-mesenchymal transition as GSK-3 phosphorylates the transcription factor Snail, targeting it for proteasomal degradation, and allowing transcription of adherens junction proteins such as E-cadherin in epithelial cells [31]. These paradoxical findings are nonetheless consistent with those of Kneidinger and colleagues, who showed that intraperitoneal administration of the GSK-3 inhibitor LiCl was capable of decreasing pulmonary collagen expression in a murine model of elastase-induced emphysema [32]. Furthermore, the selective GSK-3 inhibitor SB216763 has been demonstrated to attenuate pulmonary fibrosis induced by bleomycin [13]. In the same study it was shown that attenuation of the fibrogenic processes upon GSK-3 inhibition occurred independently of the inflammatory response, suggesting a direct effect of GSK-3 on cells regulating the fibrotic response [13,33]. In line with this contention, we have previously shown that GSK-3 inhibition or silencing of the kinase by siRNA attenuates TGF-β induced fibronectin and sm-α-actin expression in pulmonary fibroblasts [14]. In that study, pharmacological inhibition of GSK-3 by SB216763 resulted in an increase in ser133 cyclic adenosine 3′5′ monophosphate (cAMP) response element binding protein (CREB) phosphorylation in pulmonary fibroblasts, which was associated with inhibition of functional TGF-β signalling [14]. In various cells it has been demonstrated that smad-dependent signalling can be functionally antagonized by activation of CREB, which provides an explanation for the inhibitory effects of SB216763 on airway fibrosis [33-36]. Unfortunately, due to lack of availability of phospho-serine133 specific antibodies against guinea pig CREB, it was not possible to determine the phosphorylation status of CREB in our studies. Nonetheless, GSK-3 mediated regulation of CREB and smad-dependent signalling appears a plausible explanation for the paradoxical inhibition of LPS induced β-catenin expression and subsequent matrix protein production by SB216763 as we did not observe anti-inflammatory effects of SB216763 in our experiments. Growth factors, including TGF-β, regulate cellular β-catenin expression by smad mediated gene-transcription in addition to GSK-3 dependent posttranslational effects on β-catenin protein stability [37,38]. In support, TGF-β induced β-catenin expression in pulmonary fibroblasts, and this was attenuated by either SB216763 or by smad3 inhibition using SIS3 (data not shown). In further support, a recent study indicated that hyperoxia induced β-catenin expression by pulmonary vessels could be repressed by SB216763 treatment in rats [39]. Collectively, these data indicate that *in vivo* activation of β-catenin signalling is associated with an increase in the pulmonary extracellular matrix deposition, whereas selective inhibition of GSK-3 prevents this LPS-induced process.

In addition to fibrosis, increased smooth muscle content in the airways may be part of the airway remodelling, contributing to COPD pathophysiology [40]. It is important to note, that alterations in airway smooth muscle content are observed in individuals with very severe COPD only. In our guinea pig model, we did not observe alterations in smooth muscle content, as determined by sm-MHC positive area, in either the large (cartilaginous) or smaller (non-cartilaginous) airways, which is in agreement with previously published findings in this model [20]. Of interest is that smooth muscle mass did not change in response to GSK-3 inhibition either. Published findings indicate that growth factor induced inhibition of GSK-3 promotes airway smooth muscle cell proliferation and hypertrophy [22,23]. Further, airway smooth muscle growth in response to allergen exposure correlates with GSK-3 inactivation in airway smooth muscle in mice [41]. The observation that pharmacological inhibition of GSK-3 using SB216763 is not sufficient to promote airway smooth muscle growth is therefore reassuring and provides further support for the suitability of GSK-3 as a drug target.

COPD is a disease with significant extrapulmonary effects that contribute to disease severity [24]. Therefore, we investigated right ventricle size in response to repeated LPS instillation. LPS induced right ventricle hypertrophy, which was fully prevented by SB216763. This indicates that GSK-3 contributes to this pathological feature and therefore possibly to the development of pulmonary hypertension. Although investigations on the underlying mechanisms were not part of the design of the current study, it is well known that both vascular remodelling and functional changes in the vessel wall may lead to increased resistance in the pulmonary vasculature, causing pulmonary hypertension [24]. We have previously analysed vascular remodelling extensively in the LPS-challenged guinea pig [20], but consistently found no effect on the thickness of the pulmonary artery medial area and pulmonary arteriole wall area. This suggests that the ventricle remodeling is not due to pulmonary vascular remodelling, but due to functional changes in pulmonary vascular constriction, for example as a result of hypoxia [20].

Taken together, this study demonstrates that topical application of the selective GSK-3 inhibitor SB216763 is capable of preventing pulmonary remodelling effects in a guinea pig model of COPD. Although the exact mechanism(s) underlying these effects remains to be established, we propose that the anti-remodelling properties of the drug may be related to CREB dependent attenuation of smad activation. In conclusion, our findings suggest that inhibition of GSK-3 may provide a novel means for the treatment of chronic airway diseases, such as COPD.

## Competing interests

The authors declare that they have no competing interests.

## Authors’ contributions

HAB, HM, HAMK and RG designed the study, HAB, SB, KHV, MS performed experiments, HAB, HM, AMWJS, RCL, HAMK, RG analyzed and interpreted the data; HAB and RG drafted the manuscript; HM, AMWJS, RCL, HAMK critically read the manuscript and provided important intellectual content; all authors read and approved the final version of the manuscript.
